# Diagnostic Performance of ^18^F-FDG PET(CT) in Bone-Bone Marrow Involvement in Pediatric Neuroblastoma: A Systemic Review and Meta-Analysis

**DOI:** 10.1155/2021/8125373

**Published:** 2021-06-16

**Authors:** Lixin Sun, Bingye Zhang, Ruchen Peng

**Affiliations:** ^1^Department of Nuclear Medicine, Beijing Luhe Hospital, Capital Medical University, No. 82 Xinhua South Road, Tongzhou District, Beijing 101149, China; ^2^Department of Radiology, Beijing Luhe Hospital, Capital Medical University, No. 82 Xinhua South Road, Tongzhou District, Beijing 101149, China

## Abstract

**Objective:**

We sought to perform a systemic review and meta-analysis of the diagnostic performance of ^18^F-fluorodeoxyglucose (^18^F-FDG) positron emission tomography (computed tomography) (PET(CT)) in detection of bone and/or bone marrow involvement (BMI) in pediatric neuroblastoma (NB).

**Materials and Methods:**

We searched electronic databases Pubmed and Embase to retrieve relevant references. We calculated pooled sensitivity, specificity, positive and negative likelihood ratios (LR+ and LR−), diagnostic odds ratio (DOR), and the area under the curve (AUC). Moreover, a summary receiver operating characteristic (SROC) curve and likelihood ratio dot plot were plotted. Study-between statistical heterogeneity was evaluated via *I*-square index (*I*^2^). Subgroup analyses were used to explore heterogeneity.

**Results:**

Seven studies including 127 patients were involved in this meta-analysis. The overall sensitivity and specificity were 0.87 (95% CI: 0.65–0.96) with heterogeneity *I*^2^ = 88.1% (*p* < 0.001) and 0.96 (95% CI: 0.67–1.00) with heterogeneity *I*^2^ = 77.8% (*p* < 0.001), respectively. The pooled LR+, LR−, and DOR were 21.3 (95% CI: 2.1–213.9), 0.14 (95% CI: 0.05–0.40), and 157 (95% CI: 16–1532), respectively. The area under the SROC curve was 0.97 (95% CI: 0.95–0.98).

**Conclusions:**

Through a meta-analysis, this study suggested that ^18^F-FDG PET(CT) has a good overall diagnostic accuracy in the detection of bone/BMI in pediatric neuroblastoma.

## 1. Introduction

Neuroblastoma (NB) is the most common extracranial pediatric malignancy, derived from the peripheral sympathetic nervous system [1]. Approximately 50% patients presented distant metastasis to bone marrow, bone, lymph nodes, and liver [2]. Metastatic bone-bone marrow is a sign of advanced disease and considered to imply poor prognosis. Bone marrow (BM) minimal residual disease (MRD) is considered as a consistent independent prognosis factor of survival after immunotherapy [3]. Bone marrow biopsy (BMB) is currently a “gold standard” modality in identifying bone marrow involvement (BMI) due to its advantages of diagnosis, staging, and treatment monitoring in childhood malignances [4]. Nevertheless, it is a painful and invasive procedure, especially for children. Additionally, the major drawback of BMB is that it may miss focal NB tumor cells deposits [5].

Iodine-123 metaiodobenzylguanidine (^123^I-MIBG) scintigraphy is the mainstay imaging in pediatric NB. However, it is false negative in approximately 10% of tumors due to no concentration of MIBG. ^18^F-fluorodeoxyglucose (^18^F-FDG) positron emission tomography (PET) has an alternative and complementary role in these cases, even in poorly differentiated tumors [6, 7]. Previous meta-analysis suggested that ^18^F-FDG PET had a higher sensitivity but lower specificity than MIBG scintigraphy in NB lesion detection [8]. Another recent meta-analysis reported a 17% pooled sensitivity of positron emission tomography (computed tomography) (PET(CT)) for the detection of bone marrow in pediatric NB in their subgroup analysis, which was significantly lower than several studies [9–16]. Superiorities of PET are high ^18^F-FDG avidity of the bone marrow and imaging of the entire marrow [17]. Moreover, PET showed more osteomedullary abnormalities than MIBG scintigraphy and bone scan, and it could have a better definition of FDG abnormalities in bone marrow and bone with the use of PET [18]. Notably, the parameters, total lesion glycolysis (TLG) and metabolic tumor volume (MTV), were independent predictors for recurrence free survival (RFS) and/or overall survival (OS) of FDG PET/CT in pediatric NB [19, 20]. However, FDG uptake is sometimes less specific in bone marrow. Neither PET(CT) nor MIBG scintigraphy reliably detects small-volume bone marrow abnormalities [21].

The purpose of this meta-analysis is to meta-analyse the diagnostic performance of ^18^F-FDG PET(CT) in assessing bone/bone marrow involvement in pediatric NB patients to provide more evidence-based data for clinicians.

## 2. Materials and Methods

This systematic review and meta-analysis was performed according to the Preferred Reporting Items for Systematic Reviews and Meta-Analyses (PRISMA) guidelines. However, it was not registered on the international prospective register of systematic reviews (PROSPERO) [22].

### 2.1. Search Strategy

Electronic literature databases (PubMed and Embase) were searched on 1 April 2021. The search algorithm was based on the combined terms: (1) “positron emission tomography” or “PET” or “positron emission tomography-computed tomography” or “PET-CT” AND (2) “neuroblastoma” or “neuroblastomas” or ((“pediatric” or “childhood”) and (“malignance” or “solid tumor”)) AND (3) “diagnostic accuracy” or “sensitivity” or “specificity”. No beginning date or language restriction was included in our search.

### 2.2. Inclusion Criteria and Exclusion Criteria

Studies that met the following criteria were included in this analysis: (1) the main topic was the diagnostic accuracy of ^18^F-FDG PET(CT) in the detection of bone or bone marrow involvement in pediatric NB patients; (2) BMB as the gold standard; (3) sufficient data to reassess sensitivity and specificity.

The exclusion criteria were (1) articles not within the field of our study, (2) review articles, abstracts, case reports, editorial and chapters; (3) insufficient data to reassess sensitivity and/or specificity. Two researchers (L. S. and B. Z.) reviewed the full papers independently to decide which were eligible for inclusion.

### 2.3. Data Extraction and Quality Assessment

Basic information was grouped together, namely, first author's name, publication date, country, study design, and patients' characteristics, as well as technical aspects, such as scan model and injection dose. Each included study was analyzed to obtain the numbers of true positives (TP), false positives (FP), true negatives (TN), and false negatives (FN) for the detection of metastatic bone-bone marrow of NB [23]. We applied the Quality Assessment of Diagnostic Accuracy Studies 2 (QUADAS-2) tool to assess studies quality [24]. Each article was reviewed by two reviewers (L. S. and B. Z.), and discrepancies were resolved in a consensus meeting. The result was judged as true positive if PET(CT) detected the BMI on imaging and was confirmed by BMB findings (one study was confirmed by BMB or magnetic resonance imaging (MRI)).

### 2.4. Statistical Analysis

The purpose of our analysis was to calculate pooled sensitivity, specificity, positive likelihood ratio (LR+), negative likelihood ratio (LR−) and diagnostic odds ratio (DOR) with 95% confidence interval (CI) for the detection of BMI. We applied a bivariate random effects model to calculate the summary sensitivity and specificity, and then we used the same model to plot summary receiver operating characteristic (SROC) curves and evaluate the areas under the curves (AUC).

To assess study-between statistical heterogeneity, we used the *I*-square index (*I*^2^) statistics. *I*^2^ describes the proportion of the variability in effect estimates, which are due to heterogeneity rather than chance. We performed subgroup analyses to explore heterogeneity based on analysis level (lesion-based vs. patient-based) and scan model (PET vs. PET/CT). Additionally, Meta-disc 1.4 was used for subgroup analyses. Publication bias was evaluated by Deeks' test regression. Data analysis was performed by using the “Midas” module in Stata software version 15.0 (StataCorp, College Station, TX, USA). Values were considered statistically significant if the two-sided *p* value was <0.05.

## 3. Results

### 3.1. Literature Search

**Figure 1:**
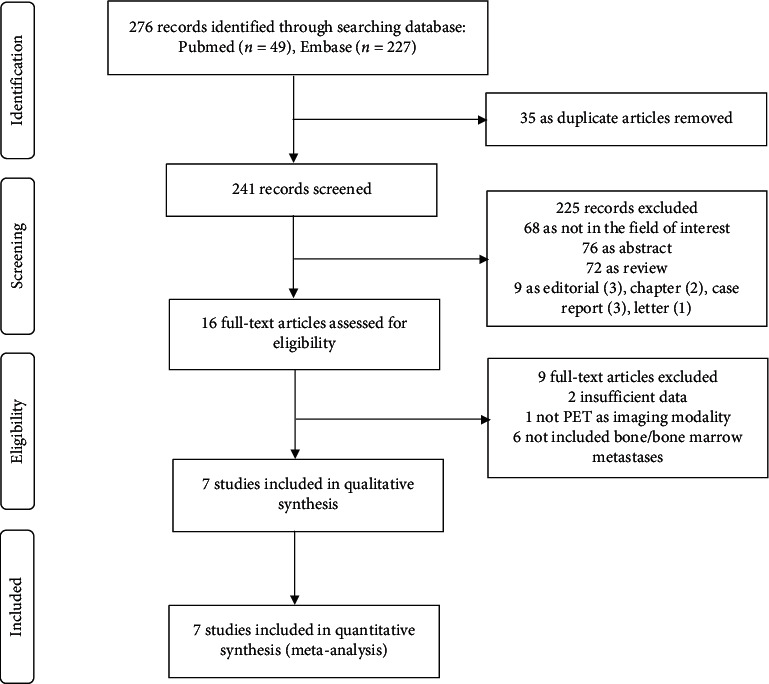
Flow algorithm of eligible literature.

Our search strategy initially identified 276 studies, among which 49 were from PubMed and 227 were from Embase. Among these, 35 duplicate articles as well as 225 studies not met the inclusion criteria were excluded. Then, 16 full-text articles were selected to read carefully, and 7 studies comprising a total of 127 patients were enrolled in the current meta-analysis finally [10–16]. A flow diagram of eligible literature is shown in Figure 1.

### 3.2. Characteristics of the Included Studies

The basic information (first author, publication year, country, and patients' characteristics) and detailed technical aspects (scan model, injection dose, interval time, and imaging analysis) of included studies are listed in Table 1. Among the seven included studies, one had a prospective and six had retrospective design. Of the seven included studies, five reported on patient-based analysis, and the rest two described on lesion-based analysis. Across the seven included studies, four studies only focused on BMI in NB, whereas the remaining three enrolled participants extended to various pediatric malignancies. Of note, only one of the seven studies confirmed BMI in NB children by BMB or MRI.

### 3.3. Quality Assessment

**Figure 2:**
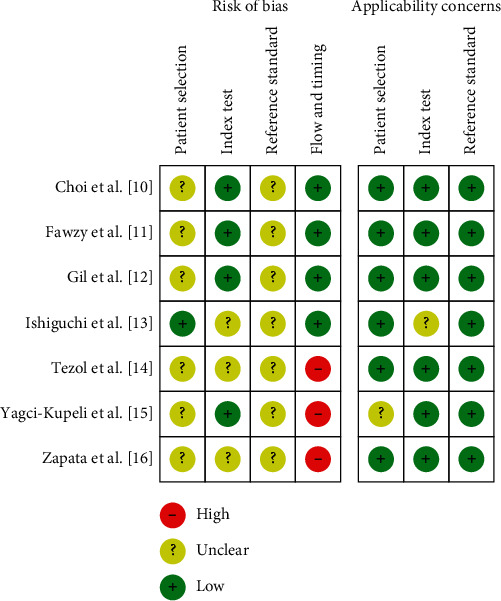
Methodological quality summary by Quality Assessment of Diagnostic Accuracy Studies 2 (QUADAS-2) tool.

Results from methodological quality analysis are summarized in Figure 2. Based on patient selection, 6 studies [10–12, 14–16] showed an unclear risk of bias owing to insufficient information of consecutive patient enrollment or time limitation. With regards to index test, 3 studies [13, 14, 16] exhibited an unclear risk of bias due to the fact that they did not mention whether or not the operators interpreted the images without reference standard. All studies [10–16] revealed an unclear risk in reference standard, owing to lack of information with regards to histological results without prior clinical and imaging data. 3 studies [14–16] revealed a high risk in flow and timing since not all of the patients were included in this meta-analysis. However, most of the studies were considered to have low applicability in the patient selection, index test, and reference standard domains [23].

### 3.4. Pooled Diagnostic Performance of PET(CT)

**Figure 3:**
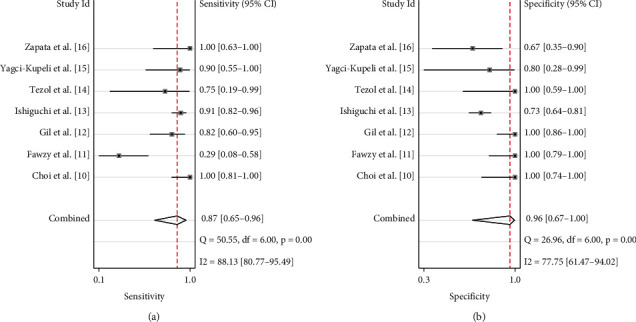
Forest plot of the summary sensitivity and specificity of F-18 fluorodeoxyglucose (^18^F-FDG) positron emission tomography (computed tomography) (PET(CT)) for the detection of bone metastases and/or bone marrow involvement (BMI) in pediatric neuroblastoma (NB) patients.

**Figure 4:**
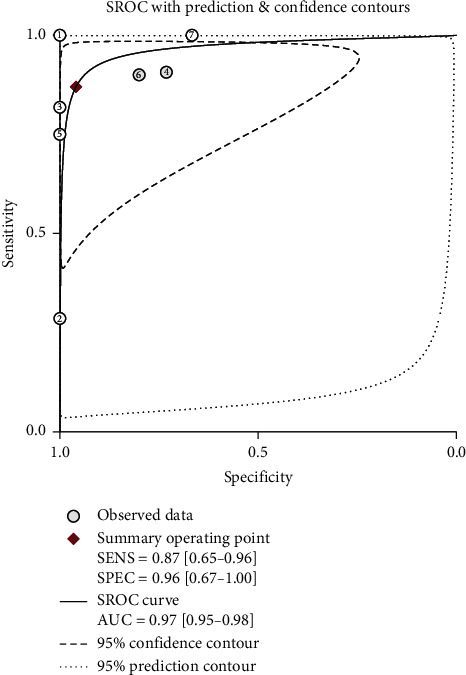
The summary receiver operating characteristic (SROC) curve of the diagnostic accuracy of ^18^F-FDG PET(CT) in detecting bone metastases and/or BMI in pediatric NB patients. The area under ROC was 0.97.

Their pooled sensitivity, specificity, LR+, LR−, and DOR of PET(CT) were 0.87 (95% CI: 0.65–0.96), 0.96 (95% CI: 0.67–1.00), 21.3 (95% CI: 2.1–213.9), 0.14 (95% CI: 0.05–0.40), and 157 (95% CI: 16–1532), respectively. We summarized sensitivities and specificities of PET(CT) for the detection of bone metastases/BMI in pediatric NB by using forest plots as shown in Figure 3. *I*^2^ values for sensitivity and specificity were 88.1% (*p* < 0.001) and 77.8% (*p* < 0.001), respectively. The SROC curve is shown in Figure 4. The area under SROC of PET(CT) was 0.97 (95% CI: 0.95–0.98).

### 3.5. Publication Bias and Subgroup Analysis

Deeks' funnel plot asymmetry test was designed to assess the possibility of publication bias. There was no significant publication bias (*p*=0.53) of PET(CT) (Figure S1). Sensitivity and specificity values were both highly heterogeneous. We performed subgroup analyses based on the factors as follows: analysis level (patient-based vs. lesion-based level) and scan model (PET vs. PET/CT). The results are listed in Table 2. However, the two factors failed to explain heterogeneity.

### 3.6. LR Scattergram

**Figure 5:**
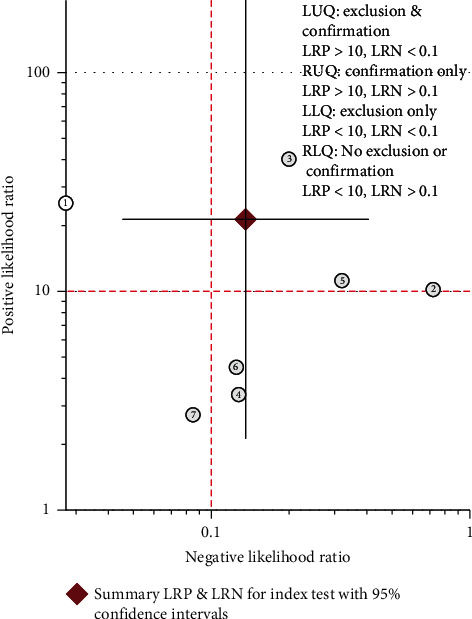
Scattergram suggesting that ^18^F-FDG PET(CT) could be useful for the confirmation of bone metastases and/or BMI in pediatric patients with NB.

Figure 5 displays the summary point of LRs in the right upper quadrant. The combined LR+ for the detection of BMI was >10, and LR− was >0.1, which suggested that ^18^F-FDG PET(CT) could provide useful information for the confirmation of BMI in pediatric NB patients.

## 4. Discussion

MIBG is an analog of norepinephrine, which is specifically taken up by norepinephrine transporters. ^123^I/^131^I MIBG scans have been widely used for diagnosis and staging NB. However, lack of these transporters as well as small and necrosis lesions easily leads to false-negative findings. As reported, the sensitivity of MIBG scan was limited in detecting single bone and bone marrow metastases [25–27]. PET/CT is the most commonly used imaging modality for staging and treatment monitoring of adult tumors. The application of PET(CT) in NB has long been studied. However, its usefulness in assessing NB is still under debate. Kushner and colleagues found that PET was equal to ^123^I/^131^I MIBG scans in detecting NB when including the skull in the comparison, but was superior in only extracranial skeletal structures [17]. Additionally, Fawzy et al. suggested overall accuracy of ^18^F-FDG PET/CT was slightly higher than MIBG in the detection of BMI with values of 66.6% and 66.3% [11]. In contrast, several studies showed that ^123^I-MIBG scan was more sensitive in assessment of bony metastatic lesions than ^18^F-FDG PET [28, 29]. Consequently, it still remains difficult to predict the clinical values of ^18^F-FDG PET in pediatric NB.

The findings of this meta-analysis revealed that ^18^F-FDG PET(CT) has high diagnostic accuracy, as evidenced by a sensitivity of 0.87 (95% CI: 0.65–0.96), a specificity of 0.96 (95% CI: 0.67–1.00), and AUC of 0.97 (95% CI: 0.95–0.98). In addition, the combined positive likelihood ratio was >10 and negative likelihood ratio was >0.1, which indicated that ^18^F-FDG PET(CT) played an useful role in the confirmation of BMI in pediatric NB patients.

Our results were significantly different from the findings of Li and colleagues [9]. Their global sensitivity, specificity, positive likelihood ratio, negative likelihood ratio, and DOR were 17% (95% CI, 9%–30%), 78% (95% CI, 61%–89%), 0.80 (95% CI, 0.29–2.20), 1.05 (95% CI, 0.82–1.35), and 0.966 (95% CI, 0.254–3.671), respectively; the AUC was 0.32 (95% CI, 0.28–0.36). The summary estimates were particularly lower than those of the present study. Of note, there are several differences between the two meta-analyses because we have a more strict inclusion criterion. First, we only included the studies providing adequate data to reassess the specificity. Second, radio-tracer was limited to ^18^F-FDG, due to its wide application. Moreover, BMB was considered as the reference standard in most of this meta-analysis. Only one study used BMB/MRI as a reference standard. To some extent, our results indicated the effectiveness of ^18^F-FDG PET(CT) in bone/bone marrow involvement in pediatric NB patients.

Subgroup analysis, about the scan model of PET and PET/CT, showed that the PET/CT has a lower diagnostic accuracy than PET. This might partly due to small number of studies. There were two studies evaluating the usefulness of FDG PET, with regard to detection of bone/BMI in pediatric NB patients. Moreover, one of the two studies focused on pretreatment patients, which reduced the number of false positives. Since it has been known that chemotherapy can stimulate hyperactivity in bone marrow, subgroup analysis performed by two different analysis types suggested that patient-based analysis showed a lower sensitivity but a higher specificity than those of lesion-based analysis. We did not perform subgroup analyses targeting study design (prospective vs. retrospective) and reference standard (BMB vs. BMB/others) because of limited number of history reports. We also failed to perform subgroup analysis on age of children since there was no consensus of median or mean age reported in the studies. However, these potential factors might be the sources of heterogeneity.

The present study has some limitations. First, the total number of involved studies was small (*n* = 7) since we only included studies reporting ^18^F-FDG PET(CT) in the detection of bone/BMI in pediatric NB and eliminated studies without specificity. Moreover, most of the studies did not have large numbers of patients or lesions, and the patients included in this meta-analysis were 127. The low number of studies and patients limited meta-regression for detecting the heterogeneity. Second, there was only one prospective study in our cases. It was difficult to avoid patient selection, interpretation, and verification bias, owing to the fact that a majority of rest were retrospective studies. For example, patient recruitment was always not consecutive; the reviewers may have known reference standard when interpreting the results of ^18^F-FDG PET and then exaggerated the diagnostic accuracy. Third, not all of the studies used BMB as the reference standard. Although BMB may exhibit potential sampling error, it is still the gold standard for BMI. Using BMB or other modalities (imaging or clinical follow-up) as a reference standard is easy to increase false-negative or false-positive findings. Fourth, among all of the studies, three were aimed at evaluating bone/BMI in various pediatric malignancies, so information of NB patients was not as detailed as other articles.

## 5. Conclusions

Summarily, the present meta-analysis suggested that ^18^F-FDG PET(CT) has a good overall diagnostic accuracy with high sensitivity, specificity, and AUC in the detection of bone or bone marrow involvement in pediatric neuroblastoma. Notably, the number of studies using ^18^F-FDG PET(CT) in the assessment of bone/BMI in NB was still small, necessitating further investigation.

## Figures and Tables

**Table 1:** Characteristic of involved studies.

Sources	Country	Study design	No.	Age	F/M	Scan model	Injection dose	Interval time	Imaging analysis	Analysis type	Stage (INSS)	Reference standard
Choi et al. [10]	South Korea	R	30	2.7 median	18/12	PET	5.18 MBq/kg	60 min	SUVmax	PB	I–IV	BMB/MRI
Fawzy et al. [11]	Egypt	P	30	3.77 mean	16/14	PET/CT	NA	NA	SUVmax	PB	III-IV	BMB
Gil et al. [12]	South Korea	R	8	3.6 median	5/3	PET	400 MBq^*∗*^	60 min	Visual	LB	III-IV	BMB
Ishiguchi et al. [13]	Japan	R	13	2.9 ± 2.0 mean	6/7	PET/CT	3.7 MBq/kg	NA	Visual	LB	IV	BMB
Tezol et al. [14]	Turkey	R	11	2.3 ± 1.6 mean	NA	PET/CT	NR	60 min	Visual	PB	NA	BMB
Yağcı-Küpeli et al. [15]	Turkey	R	15	4 median	7/8	PET/CT	185 MBq	60 min	Visual	PB	NA	BMB
Zapata et al. [16]	USA	R	20	3.8 mean	12/8	PET/CT	NA	NA	Visual	PB	NA	BMB

Note: NA—not available, R—retrospective, P—prospective, No.—number of included patients, ^*∗*^—the max injection dose, SUVmax—maximum standard uptake value, LB—lesion-based, PB—patient-based, INSS—International Neuroblastoma Staging System, BMB—bone marrow biopsy, MRI—magnetic resonance imaging.

**Table 2:** Subgroup analyses.

Characteristic (studies)	Sensitivity (95% CI)	Specificity (95% CI)	LR+ (95% CI)	LR− (95% CI)	DOR (95% CI)	AUC
Analysis						
LBA (2)	0.89 (0.81–0.94)	0.78 (0.70–0.85)	8.67 (0.57–130.98)	0.15 (0.09–0.27)	43.78 (7.74–247.59)	NA
PBA (5)	0.78 (0.64–0.88)	0.90 (0.79–0.97)	5.25 (1.91–14.44)	0.18 (0.03–1.07)	43.33 (10.12–185.47)	0.94

Scan model						
PET (2)	0.90 (0.76–0.97)	1.00 (0.90–1.00)	31.82 (4.60–220.10)	0.11 (0.02–0.77)	348.28 (31.97–3794.50)	0.50
PET/CT (5)	0.83 (0.75–0.89)	0.77 (0.69–0.84)	3.38 (2.54–4.51)	0.22 (0.05–0.99)	26.61 (12.31–57.54)	0.90

## Data Availability

All analyses were based on previously published studies. Hence, data sharing is not applicable to this article.

## Supplementary Materials

Supplementary Materials
*Figure S1*. Deeks funnel plot asymmetry test for publication bias of ^18^F-FDG PET(CT) in the detection of bone/bone marrow involvement in pediatric NB.
