# Supplementary figures and images for: Diagnostic Performance of 18F-FDG PET(CT) in Bone-Bone Marrow Involvement in Pediatric Neuroblastoma: A Systemic Review and Meta-Analysis
Source: Contrast Media Mol Imaging. 2021 Jun 16;2021:8125373. doi: 10.1155/2021/8125373 (PMC8221854; doi:10.1155/2021/8125373)

Deeks' Funnel Plot Asymmetry Test  
pvalue = 0.53

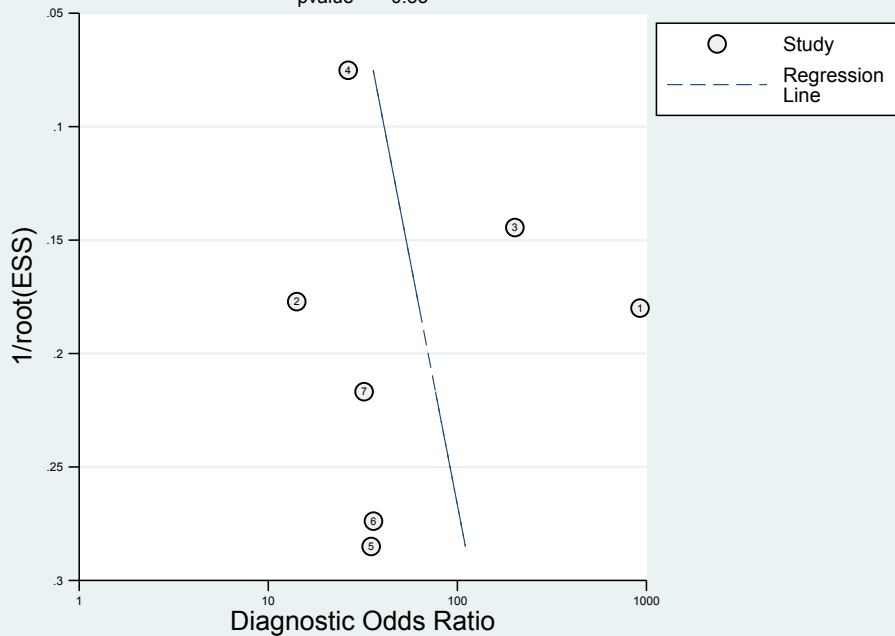

Supplement: Supplementary Materials — Figure S1. Deeks funnel plot asymmetry test for publication bias of 18F-FDG PET(CT) in the detection of bone/bone marrow involvement in pediatric NB. [file 8125373.f1.zip › 8125373.f1/Supplememt Figure 1.pdf]
